# Atom Probe Tomography Analysis of TiC*_x_* Powders Synthesized by SHS in Al/Fe/Cu–Ti–C Systems

**DOI:** 10.3390/ma12244095

**Published:** 2019-12-07

**Authors:** Shenbao Jin, Haokai Su, Gang Sha

**Affiliations:** Herbert Gleiter Institute of Nanoscience, School of Materials Science and Engineering, Nanjing University of Science and Technology, Nanjing 210094, China; jinshenbao@njust.edu.cn (S.J.);

**Keywords:** titanium carbide, stoichiometry, self-propagating high-temperature synthesis, atom probe tomography

## Abstract

The stoichiometry of titanium carbide (TiC*_x_*) particles is important in determining particle properties. Spherical TiC*_x_* powders with particle sizes of 1–5 μm were produced by self-propagating high-temperature synthesis (SHS) in 30 wt.% Al–, 30 wt.% Cu–, and 30 wt.% Fe–Ti–C systems, respectively. To measure the compositions of the carbide powders, atom probe tomography (APT) tip specimens were carefully prepared by using a focus ion-beam milling method. APT analysis revealed that the TiC*_x_* particles formed in Al–, Cu–, and Fe–Ti–C systems are highly substoichiometric. The results are consistent with observations of the TiC*_x_* particles with a high content of oxygen and a certain amount of secondary metallic elements (Al, Cu, and Fe).

## 1. Introduction

Titanium carbide (TiC*_x_*) has been widely used for manufacturing hard alloys, wear resistance tools, and carbide steels due to its unique properties, such as its high melting point, extreme hardness, low density, good thermal conductivity, and high resistance to corrosion [1,2,3,4,5,6,7]. These properties originate from the mixed covalent-ionic-metallic bonding in TiC*_x_*, while the latter is controlled by its chemical composition. According to Massalski et al. [8], the stoichiometry (*x*) of TiC*_x_* changes in a wide range from ≈0.47 to 0.98. With increases to its stoichiometry, both modulus and hardness of TiC*_x_* increase [9,10,11,12], but the wettability with metals decreases [13,14,15]. The incorporation of impurity elements such as O and N into the TiC*_x_* particles during fabrication is also effective in influencing the properties [16,17].

The TiC*_x_* stoichiometry was found to change greatly under different fabrication conditions. Self-propagating high-temperature synthesis (SHS) as a highly time- and energy-efficient method has been widely used in producing ceramic carbides. Ti–C is a typical reaction system to fabricate TiC*_x_* through SHS. The combustion temperature increases drastically during SHS and generally exceeds 2500 K because of the highly exothermic reaction of Ti + C → TiC. Secondary metallic elements such as Al, Cu, Fe or Ni are usually added in the reactants to control the combustion temperature, and at the same time, to provide a liquid environment for the growth of TiC*_x_* particles. Yang et al. [18,19,20] found that TiC*_x_* stoichiometry could be altered greatly by adding these secondary metallic elements in the Ti-C system during SHS. In their work, the stoichiometry of TiC*_x_* was estimated by fitting the relationship of lattice parameters and stoichiometry with lattice parameters measured by using X-ray diffraction (XRD). Although the indirect measurement method has been extensively used in measuring TiC*_x_* composition, its measurement result can be highly affected by uncertainties in determining lattice parameter and the lattice parameter–stoichiometry relationship.

Conventional analytical techniques such as the wave-length dispersive X-ray (WDX) or energy dispersive X-ray (EDX) methods have also been employed to reveal chemical information of the TiC*_x_*. However, they are hardly able to provide accurate quantitative measurements, because C, O, and N as contaminants often exist in the analysis environment. Atom probe tomography (APT) is very attractive because it is powerful in detecting both light (e.g., carbon, oxygen) and heavy (e.g., titanium) elements with high sensitivity. Laser-pulsing atom probes, in particular, are capable of analyzing materials with poor conductivities, including ceramics and carbides. Carbides in steels have been investigated extensively to understand their nucleation and growth as well as hydrogen-trapping effect [21,22,23,24,25,26,27,28]. To date, only limited work has been done using APT to reveal the chemical composition of carbide powders. Weidow and Andrén et al. have systematically studied the chemistry of doped-WC powder and revealed interface segregation in sintered WC-Co composites using APT [29,30,31,32,33,34]. However, to our knowledge, no APT analysis has been done on the TiC*_x_* powders synthesized by SHS.

In the present work, APT is employed to analyze the TiC*_x_* particles synthesized in Al–, Cu–, and Fe–Ti–C systems by SHS. By analyzing APT tip specimens directly prepared from the TiC*_x_* particles (powder sample), this research aims to unveil the composition of the TiC*_x_* particles and investigate the effect of the second metallic elements of Al, Cu, and Fe on carbide’s chemical composition.

## 2. Experimental Methods

### 2.1. SHS Experiments and the Synthesized TiC_x_ Powders

**Figure 1:**
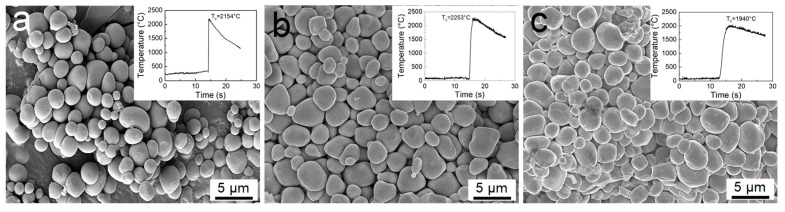
Typical morphologies of titanium carbide (TiC*_x_*) particles formed in (**a**) Al–Ti–C, (**b**) Cu–Ti–C, and (**c**) Fe–Ti–C systems, and the corresponding combustion temperature curves.

TiC_x_ powders were made from commercial powders of Al (>99.8% purity, ≈48 μm), Cu (>99.8% purity, ≈48 μm), Fe (>99.5% purity, ≈48 μm), Ti (>99.5% purity, ≈25 μm), and multi-walled carbon nanotubes (20 to 30 nm in diameter and approximately 30 μm in length, purity >95 wt.%) by using the SHS process. The carbon nanotubes and Ti powders were mixed together at a molar ratio of 1:1 with an addition of Al, Cu, or Fe powders in a relative quantity of 30 wt.%. Details of the SHS experimental apparatus and procedure can be found in Ref. [35]. The TiC*_x_* particles made in each Al/Cu/Fe-Ti-C system exhibit spherical or near-spheroidal morphology, as shown in Figure 1, with insets showing the corresponding combustion temperature curves. The sizes of TiC*_x_* particles in different systems are similar in the range of 1 to 5 μm. The maximum combustion temperatures for Al–, Cu–, and Fe–Ti–C systems are ≈2154, ≈2253, and ≈1940 °C, respectively.

### 2.2. XRD Analysis

Phase identification of the reacted samples was performed by using X-ray diffraction on a Bruker-AXS D8 Advance (Karlsruhe, Germany) with Cu *K_α_* radiation (λ = 0.154 nm) where the angle precision (∆θ) was in ±0.0001°, with a step of 0.02° and a counting time of 0.2 s. Afterward, the bulk samples were dissolved in an 18 vol.% HCl-distilled water solution or saturated FeCl_3_ water solution to remove the Al, Fe, and Cu coating on the TiC*_x_* particles. Lattice parameter determinations were then obtained by slow X-ray scanning of the extracted TiC*_x_* powders with a step of 0.02° and a counting time of 1 s. The error margin for the yielded lattice parameters (∆*d/d*) can be estimated according to the Bragg equation: ∆*d/d* = −∆θ/tanθ + ∆λ/λ. The results indicate that lattice parameters can be accurate to five decimal places in nanometers.

### 2.3. APT Specimen Preparation

**Figure 2:**
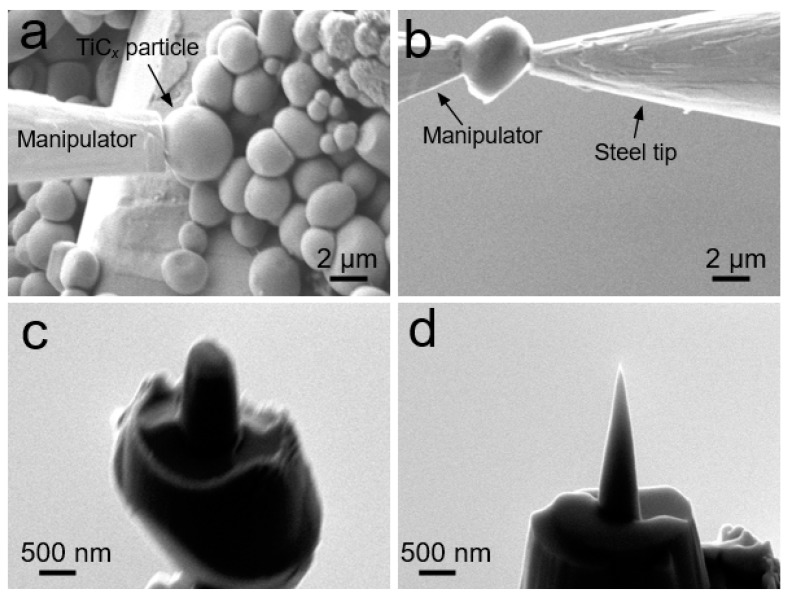
Sequence of SEM images showing atom probe specimen preparation from TiC*_x_* particles. (**a**) pick-up of one particle using the manipulator, (**b**) particle transfer from the manipulator to a steel tip, and (**c**) and (**d**) annular milling for the final tip.

APT tip specimens prepared from TiC*_x_* powders were performed using a Zeiss Auriga dual beam focused ion beam/scanning electron microscope, with procedures shown in Figure 2. First, one particle was taken out by attaching it to a manipulator from one side, as seen in Figure 2a. Then, the particle was transferred from the manipulator to a steel tip made by electro-polishing (Figure 2b). The final tip specimen was produced by annular milling to the ideal depth of each powder particle, as shown in Figure 2c,d. The method is easy to operate, and especially suitable for handling the particles with sizes less than 5 μm. In the present work, all the APT tip specimens were produced for the TiC*_x_* particles with sizes of ≈2.0 μm.

### 2.4. APT Experiment and Data Analysis

APT experiments were performed in a Local Electrode Atom Probe (LEAP4000X Si) (Madison, WI, USA) with a total detection efficiency of ≈55%, under UV laser-pulsing at a specimen temperature of 40 K, a pulse repetition rate of 200 kHz, a target evaporation rate of 0.3%, and a high laser energy of 100 pJ to avoid premature fracture during APT analysis. Reconstruction and visualization of APT datasets were performed using the Integrated Visualization and Analysis Software (IVAS 3.8.4) developed by Cameca Scientific Instruments. More detailed information about APT data acquisition settings can be found in Table S1, Supplementary Materials.

According to Angseryd et al. [36], the detection of carbon mainly suffers from two problems during APT analysis on the Ti (C,N) ceramic—considerable C-loss caused by the dead time effect for the $C_{1}^{2+}$ and $C_{1}^{+}$ ions at 6 Da and 12 Da, respectively [36,37], and C-underestimation caused by the overlap of molecular ions of $C_{2}^{+}$ (and/or $C_{4}^{2+}$) with ${\mathrm{Ti}}_{1}^{2+}$ at 24 Da since this peak has been totally assigned to ${\mathrm{Ti}}_{1}^{2+}$. More accurate C content can be grained after two steps of correction according to Ref. [36]. In the first step, the ion counts at 6 Da and 12 Da can be corrected by natural abundance ratio of ^12^C to ^13^C, which is called ^13^C-correction. In the second correction step, the C-loss caused by the overlap at 24 Da can be corrected through manual decomposition (called 24 Da-correction), of which the detailed procedures can be found in Ref. [36]. In the present work, these two correction procedures were used during data analysis.

## 3. Results and Discussion

**Figure 3:**
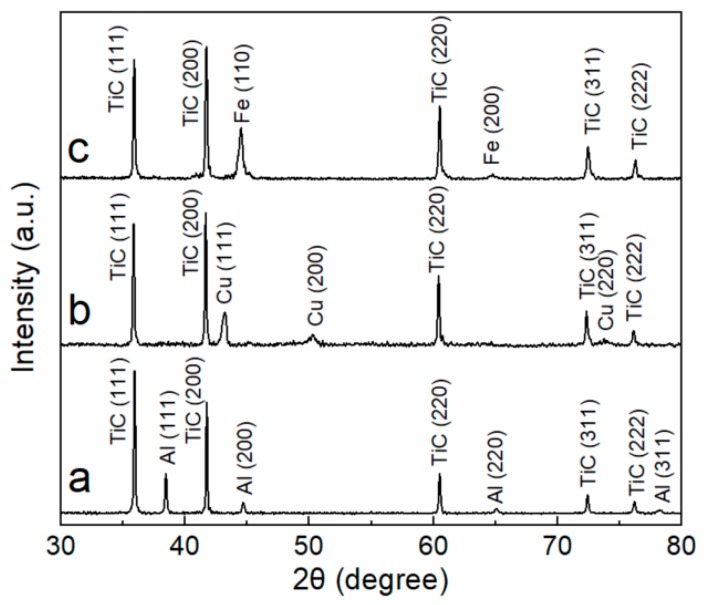
XRD results of combustion reaction products of (**a**) Al–Ti–C, (**b**) Cu–Ti–C, and (**c**) Fe–Ti–C systems.

**Figure 4:**
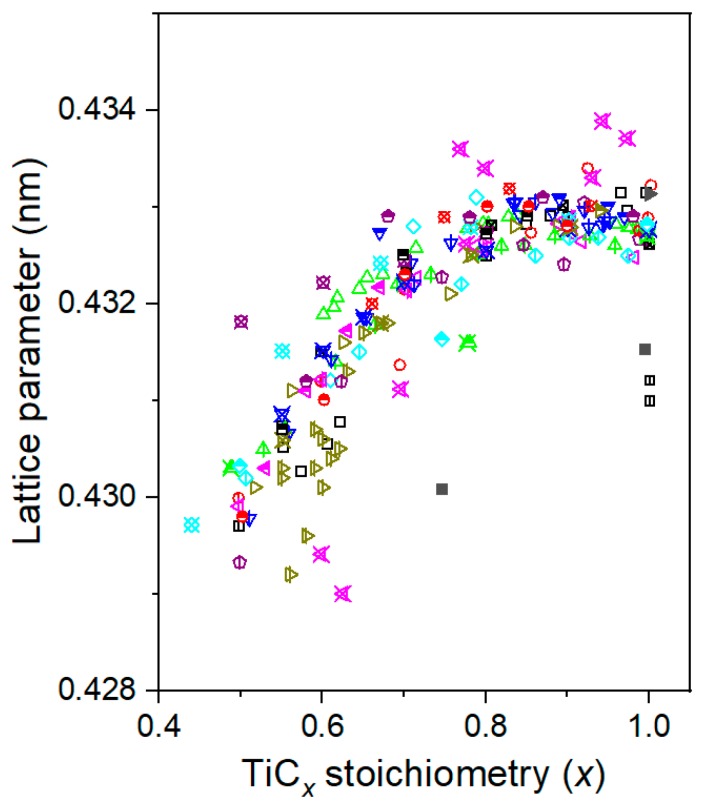
Relationship between TiC*_x_* stoichiometry and the lattice parameter reported previously.

Figure 3 shows the XRD results of TiC*_x_* powders made in Al–, Cu–, and Fe–Ti–C systems by SHS. Clearly, only Al/Cu/Fe and TiC*_x_* were detected in the combustion products, indicating that the combustion reaction was fully completed. The lattice parameters of the TiC*_x_* particles formed in Al–, Cu–, and Fe–Ti–C systems were determined as 0.43313, 0.43315, and 0.43307 nm, respectively, based on the slow XRD scanning of the TiC*_x_* powders. The average TiC*_x_* stoichiometry was then deduced based on the TiC*_x_* lattice parameter–stoichiometry relationship reported in [38], with ≈0.826, ≈0.851, and ≈0.794 for the TiC*_x_* particles in Al–, Cu–, and Fe–Ti–C systems, respectively. It is worth mentioning that the relationship between stoichiometry and lattice parameters given in Ref. [38] is a fitting result of the few experimental data obtained. For example, the strong affinity between titanium and oxygen makes oxygen prone to incorporate into the TiC*_x_* lattice during high-temperature preparation processes, and the lattice parameter of TiC*_x_* was found to change with the level of the oxygen in the carbides [39]. Further extensive experimental and simulation data in Refs. [40,41,42,43,44,45,46,47,48,49,50,51,52,53,54,55,56,57,58,59,60,61,62,63,64,65,66,67,68,69,70,71] added to the relationship plot exhibit wide scattering, as shown in Figure 4. In fact, many other factors, in addition to C/Ti stoichiometry, have been found to influence the lattice parameter of TiC*_x_*. Failure to take into account the effects of all these factors makes it difficult to obtain an accurate estimation on the C/Ti stoichiometry of these TiC*_x_* powders using the fitting method.

**Figure 5:**
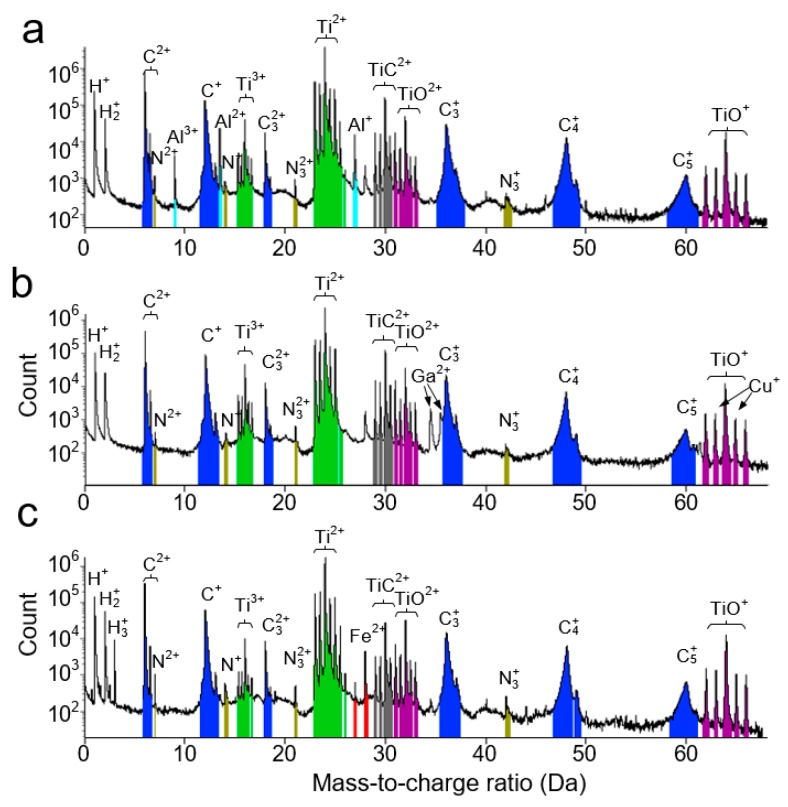
Mass spectra of the TiC*_x_* particles synthesized from combustion reaction of (**a**) Al–Ti–C, (**b**) Cu–Ti–C, and (**c**) Fe–Ti–C systems.

Figure 5 shows the APT mass spectra of the tips of the TiC*_x_* particles synthesized from Al–, Cu–, and Fe–Ti–C systems, with identified peaks labelled properly. Carbon was field-evaporated as molecular ions of $C_{1}$, $C_{2}$, $C_{3}$, $C_{4}$, and $C_{5}$. Two correction procedures were implemented in measuring the carbon content of the carbides. It should be noted that the ion count at 12 Da obtained by ^13^C-correction is only from $C_{1}^{+}$. Although $C_{2}^{2+}$ may also contribute to the peak at 12 Da, the real contribution should be quite low since no peak at 12.5 Da belonging to (^12^C^13^C)^2+^ was found in the mass spectrums. Moreover, the ion count at 24 Da obtained by 24 Da-correction can result from two different ionic species, namely, $C_{2}^{+}$ and $C_{4}^{2+}$. The latter was suggested to be the majority at 24 Da according to Ref. [72]. Considering that a relatively high laser-pulsing energy of 100 pJ (which promotes the formation of high-order combined molecular ions) was used in this experiment, the ion count at 24 Da obtained by 24 Da-correction has been completely assigned to $C_{4}^{2+}$ in this work. Table 1 lists the compositions of the TiC*_x_* particles formed in the three systems. As indicated, the C concentrations of the TiC*_x_* particles formed in Al–, Cu–, and Fe–Ti–C systems are ≈39.2, ≈40.6, and ≈34.8 wt.%, respectively. The TiC*_x_* stoichiometry values of these TiC*_x_* particles were calculated accordingly.

**Figure 6:**
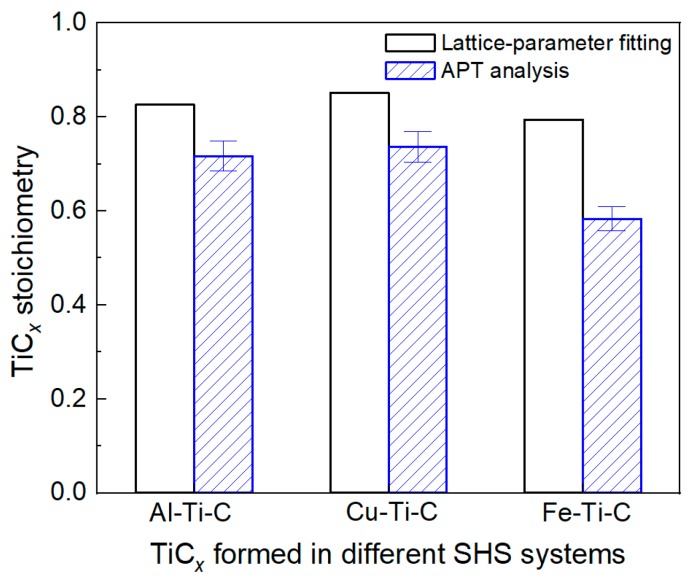
Comparison of TiC*_x_* stoichiometry values obtained by lattice-parameter fitting and APT analysis.

Figure 6 shows a direct comparison of the TiC*_x_* stoichiometry values obtained by the lattice-parameter fitting method and APT analysis. As indicated, the stoichiometric values calculated from APT for the TiC*_x_* formed in Al–/Cu–/Fe–Ti–C systems are 0.71 ± 0.03, 0.74 ± 0.03, and 0.58 ± 0.03, respectively. Note that the C/Ti stoichiometry obtained by APT analysis is lower than that obtained by the lattice-parameter fitting method. The difference may be correlated with the uncertainties in using the fitting method as mentioned previously, such as the effect of the presence of impurities in the carbides. The composition measurement results in Table 1 indicate that a high content (≈1.8 to 3.1 wt.%) of O exists in the TiC*_x_* particles synthetized by SHS. They may come from the oxidization film on the reactant powder surfaces and also the atmosphere. Although the reaction proceeded under argon gas protection, oxidization is still unavoidable because of the strong affinity between Ti and O. It has been reported that the lattice parameter decreases when O atoms are substituted for the existing C in the lattice, while the lattice parameter increases when O atoms take positions of the C-vacancies [39]. Here, the lattice substitution probably occurred since the TiC*_x_* formed during SHS exhibited highly substoichiometric properties. The TiC*_x_* stoichiometry values obtained by the lattice-parameter fitting method could be overestimations.

In addition to O and N, certain amounts of Al (≈0.48 wt.%) and Fe (≈0.18 wt.%) were also detected in the TiC*_x_* particles formed in Al– and Fe–Ti–C systems, respectively. It should be noted that the peaks at ≈28 Da should be from $N_{2}^{+}$ or Fe^+^ ions. Since $N_{3}^{+}$ and $N_{3}^{2+}$ have been found at 42 Da and 21 Da, respectively, it is reasonable to believe that $N_{2}^{+}$ ions should also exist. On the other hand, the reactant powders were homogeneously mixed by low-speed ball milling before combustion synthesis, and steel balls were used in that process. Therefore, slight Fe impurity may be induced in the reactant powders after ball milling and enter into the TiC*_x_* products. The contents of 28 Da ions in TiC*_x_* formed in Al–/Cu–/Fe–Ti–C systems are ≈0.052, ≈0.063, and ≈0.181 wt.%, respectively. Clearly, the partition of Fe is evident in the TiC*_x_* particles formed in the Fe–Ti–C system. The solubility of these secondary metallic elements in TiC*_x_* is quite low at low temperatures. Therefore, they are believed to have been incorporated into the TiC*_x_* lattice in the fast growth stage at high temperatures, and then to have remained during cooling. The solubility of these impurities is actually related to the carbon vacancies in TiC*_x_*. It has been found that considerable solubility occurred only when a very large number of carbon vacancies were present [73], which confirms that the TiC*_x_* particles formed here during SHS are highly substoichiometric. According to [74], Al is quite effective in reducing the twin boundary energy of TiC*_x_* and thus promotes the formation of stacking faults and microtwins during the growth. In [74], Al is a real impurity, and is only present in several TiC*_x_* particles in a local area. Our APT results, on the other hand, indicate that the Al and Fe distribution seems to be quite uniform in the TiC*_x_* particles (see Figure S1 in Supplementary Materials). First-nearest neighbor distance distribution (NND) of Al and Fe atoms was done in APT reconstructions of TiC*_x_* formed in Al– and Fe–Ti–C systems (Figure S2, Supplementary Materials). As indicated, the experimental NND curves of Al and Cu are coincident with the randomized curves, which confirms that the distribution of Al and Fe in the TiC*_x_* particles is uniform. The existence of these secondary metallic elements also influences the lattice parameters of the TiC*_x_* and thus the stoichiometry measurement through the lattice-parameter fitting method. In contrast to Al and Fe, the Cu content in the synthesized TiC*_x_* particles is quite low (i.e., ≈0.01 wt.%), suggesting that the solubility of Cu in TiC*_x_* is negligible even at high temperatures.

Also from Figure 6, the concentration of carbon and thus the C/Ti stoichiometry in TiC*_x_* formed in the Al–Ti–C and Cu–Ti–C systems are higher than that formed in the Fe–Ti–C system. In the former two systems, once C atoms dissolve into the Al–Ti or Cu–Ti melt, they react with Ti immediately because of extremely low solubility. In contrast, the solubility of C in Fe is much higher, especially at higher temperatures. Therefore, a considerable number of carbon atoms stay in the Fe matrix during a high-temperature reaction process. The results are consistent with [19], that the Fe addition can decrease the stoichiometry of TiC*_x_* by the formation of Fe–C solid solutions.

## 4. Conclusions

Spherical TiC*_x_* powders with particle sizes of 1–5 μm were prepared by self-propagating high-temperature synthesis in 30 wt.% Al–, 30 wt.% Cu–, and 30 wt.% Fe–Ti–C systems with reactant molar ratios of C/Ti = 1.0. The composition of the TiC*_x_* particles was revealed by atom probe tomography.

It was found that the TiC*_x_* particles formed in Al–, Cu–, and Fe–Ti–C systems are highly substoichiometric. Moreover, the concentration of carbon and thus the C/Ti stoichiometry in TiC*_x_* formed in the Al–Ti–C and Cu–Ti–C systems are higher than that formed in the Fe–Ti–C system. The substoichiometric nature enables these SHS-synthesized TiC*_x_* particles to have an improved wettability and thus a higher interfacial affinity with metals, expanding thereby their application as reinforcements in metal-based composites and grain refiners during the casting of metals and alloys.

During SHS, ≈0.48 wt.% Al and ≈0.18 wt.% Fe exist in the TiC*_x_* particles synthetized in Al– and Fe–Ti–C systems, respectively. In contrast, only ≈0.01 wt.% Cu exists in the TiC*_x_* particles synthesized in the Cu–Ti–C system, suggesting that the solubility of Cu in TiC*_x_* is negligible even at high temperatures.

## Figures and Tables

**Table 1:** Composition measured by atom probe tomography (APT) analysis of the TiC*_x_* particles formed in Al–, Cu–, and Fe–Ti–C systems. Three correct methods were used successively: (1) peak decomposition in IVAS software (Decomp.); (2) ^13^C correction (13C_Corr_); and (3) correction of the peak at 24 Da (24Da).

	Ti	C	O	N	Al	Cu	Fe
TiC*_x_* in Al–Ti–C	57 ± 1.0	39 ± 1.1	3.12 ± 0.06	0.073 ± 0.001	0.48 ± 0.01	-	-
TiC*_x_* in Cu–Ti–C	58 ± 1.0	41 ± 1.1	1.86 ± 0.01	0.052 ± 0.001	-	0.012 ± 0.001	-
TiC*_x_* in Fe–Ti–C	62 ± 1.0	35 ± 1.0	2.41 ± 0.04	0.185 ± 0.003	-		0.18 ± 0.01

## Supplementary Materials

The following are available online at https://www.mdpi.com/1996-1944/12/24/4095/s1. Figure S1: Atom maps of APT tips of TiC*_x_* particles synthesized from combustion reaction of (**a**) Al–Ti–C, (**b**) Cu–Ti–C and (**c**) Fe–Ti–C systems. As indicated by mass spectrum results in Figure 5, the Cu+ peaks overlap with TiO+ peaks at 63 Da and 65 Da. Although the Cu content can be obtained by preforming peak decomposition using IVAS software, its distribution is indistinguishable from that of TiO. Therefore, the Cu atoms map is not showed here, Figure S2: First-nearest neighbor distance distribution (NND) of (**a**) Al and (**b**) Fe atoms in APT reconstructions of TiC*_x_* formed in Al– and Fe–Ti–C systems, respectively, Table S1: Atom Probe Tomography Data Acquisition Settings and Data Summary, Table S1: Atom probe tomography data acquisition settings and data summary.
