# Supplementary material for: Atom Probe Tomography Analysis of TiCx Powders Synthesized by SHS in Al/Fe/Cu–Ti–C Systems
Source: Materials (Basel). 2019 Dec 7;12(24):4095. doi: 10.3390/ma12244095 (PMC6947562; doi:10.3390/ma12244095)
Supplement: Supplementary file 1 [file materials-12-04095-s001.pdf]

materials

Supplementary Materials

Atom Probe Tomography Analysis of TiC_x Powders Synthesized by SHS in Al/Fe/Cu–Ti–C Systems

Shenbao Jin, Haokai Su and Gang Sha *

Herbert Gleiter Institute of Nanoscience, School of Materials Science and Engineering, Nanjing University of Science and Technology, Nanjing 210094 China; jinshenbao@njust.edu.cn (S.J.); 117116022381@njust.edu.cn (H.S.)

* Correspondence: gang.sha@njust.edu.cn

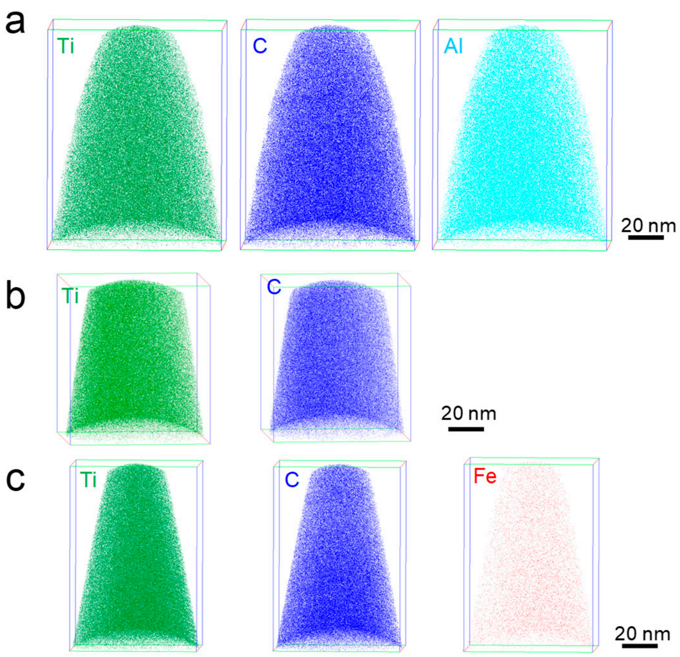

Figure S1. Atom maps of APT tips of TiC_x particles synthesized from combustion reaction of (**a**) Al–Ti–C, (**b**) Cu–Ti–C and (**c**) Fe–Ti–C systems. As indicated by mass spectrum results in Figure 5, the Cu+ peaks overlap with TiO+ peaks at 63 Da and 65 Da. Although the Cu content can be obtained by preforming peak decomposition using IVAS software, its distribution is indistinguishable from that of TiO. Therefore, the Cu atoms map is not showed here.

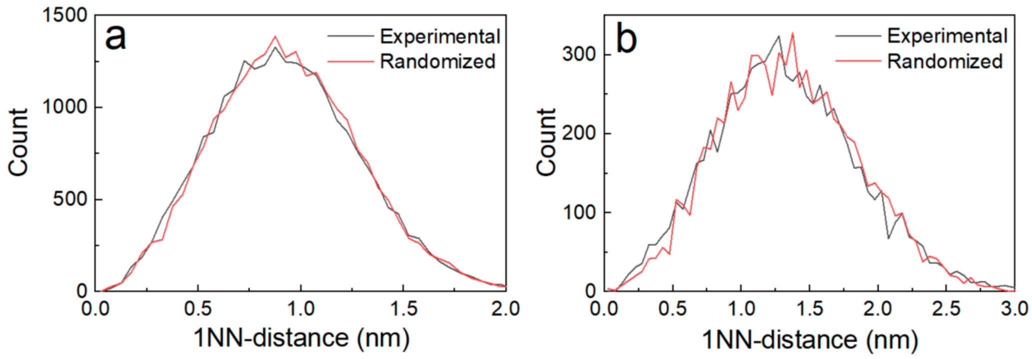

Figure S2. First-nearest neighbor distance distribution (NND) of (**a**) Al and (**b**) Fe atoms in APT reconstructions of TiC_x formed in Al– and Fe–Ti–C systems, respectively.

Table S1. Atom Probe Tomography Data Acquisition Settings and Data Summary.

Specimen/Data Set	TiC_x in Al–Ti–C	TiC_x in Cu–Ti–C	TiC_x in Fe–Ti–C
Instrument Model	LEAP 4000X Si	LEAP 4000X Si	LEAP 4000X Si
Instrument settings			
Laser wavelength (nm)	355	355	355
Laser pulse energy (pJ)	100	100	100
Voltage pulse fraction (%)	20	20	20
Pulse frequency (kHz)	200	200	200
Evaporation control	Detection rate	Detection rate	Detection rate
Target detection rate (ions/pulse)	0.003	0.003	0.003
Nominal flight path (mm)	90	90	90
Set point temperature (K)	40	40	40
Sample temperature (K)	44.4	44.4	44.4
Chamber pressure (Torr)	4.2×10^{-11}	4.2×10^{-11}	4.2×10^{-11}
Data summary	36663240	13426602	15666754
LAS root version	15.41.342j	15.41.342j	15.41.342j
CAMECAROOT version	18.46.452	18.46.452	18.46.452
Analysis software	IVAS 3.8.4	IVAS 3.8.4	IVAS 3.8.4
Total ions:	33572447	8151033	12320240
Single (%)	73.1	72.6	77.8
Multiple (%)	26.6	27.1	21.8
Partial (%)	0.3	0.3	0.3
Reconstructed ions:	33572447	8151033	12320240
Ranged (%)	91.6	89.5	89.8
Unranged (%)	8.4	10.5	10.2
Volt./bowl corr. peak (Da)	24	24	24
Mass calib. (peaks/interp.)	4/Lin.	4/Lin.	4/Lin.
(M/ΔM) for $^{48}Ti^{2+}$	651.9	685.6	702.9
Time-independent background (ppm/ns)	22.809	28.788	27.389
Reconstruction			
Final specimen state	Fracture	Fracture	Fracture
Pre-/post-analysis imaging	SEM/None	SEM/None	SEM/None
Radius evolution model	"Voltage"	"Voltage"	"Voltage"
Field factor (*k*)	3.3	3.3	3.3
Image compression factor	1.65	1.65	1.65
Assumed E-field (V/nm)	26	26	26
Detector efficiency (%)	55	55	55
Avg. atomic volume (nm³)	0.0177	0.0177	0.0177
$V_{initial}$; V_{final} (V)	2927; 6524	3488; 5612	2883; 4973

C correction method in Ref. 36 (mainly quoted from Ref. 36)

Two procedures were included to correct C content. The first procedure targets the $^{12}C^{2+}$ and $^{12}C^{+}$ peaks, while the second procedure focuses on the overlap between $^{12}C^{2+}$ and $^{48}Ti^{2+}$ at 24 Da.

Correction of $^{12}C^{2+}$ and $^{12}C^{+}$ by $^{13}C^{2+}$ and $^{13}C^{+}$ (^{13}C-correction)

The probability to detect two ^{12}C from the same pulse is more than 8000 times higher than the probability to detect two ^{13}C. Hence, the $^{13}C^{n+}$ peaks are virtually unaffected by the detector dead-time, while significant losses of $^{12}C^{n+}$ are expected. As a result, a correction (^{13}C-correction) based on the concentration of $^{13}C^{n+}$ was used. Therefore, the concentration of $^{12}C^{n+}$ was thus obtained by multiplying the measured $^{13}C^{n+}$ peaks by 92.5 ± 7.6 (the natural abundance of ^{13}C is 1.07 ± 0.08%). Since the statistical counting error is proportional to $1/\sqrt{N}$, having the analyses based on a peak that is ~1% of the main peak gives a ~10 times larger counting uncertainty. Thus, for phase analysis the uncertainty can be rather low if the dataset contains a few million atoms. However, in addition to the counting error there is an uncertainty due to the variation in ^{13}C abundance in different terrestrial sources, which is rather large. Thus, in many cases this uncertainty dominates over the counting

error. Therefore, the systematic uncertainties for the ^{13}C-correction originates from the uncertainty in natural abundance, 1.07 ± 0.08%.

Correction method for the 24 Da peak (24 Da-correction)

As a major peak 48 Ti^{n+} is expected to suffer, to some extent, from a loss due to the detector dead-time. Therefore, peak decomposition at 24 Da according to the natural abundance of Ti will always yield too little $^{12}C^{2+}$. Counts in the respective peaks of the single event spectra, $(^{46}Ti^{2+})_s$, $(^{47}Ti^{2+})_s$ and $(24\ Da)_s$, were extracted and collected. These two isotopes were used because they are not affected by interference with other peaks such as titanium hydrides. It was assumed that if the 24 Da peak had not suffered from missing ions due to dead-time issues of the detector, the multiple events should exhibit the same abundance distribution between these three peaks as the single events. Therefore, by using Eq. (S1), the amount of ions that should occur as multiple events at 24 Da could be calculated and the corrected counts of multiple events at 24 Da $(24\ Da)_{c\text{-}m}$ were obtained. The difference between the corrected counts of multiple events and the recorded multiple counts gave an estimate of the amount of ions missed by the detector, as shown in Eq. (S2). The systematic errors in the correction of the peak at 24 Da originate from the uncertainties in the data processing when manually summing up the counts, with the additional uncertainties in the ^{13}C-correction included.

$$(24Da)_{c\text{-}m}=(24Da)_s\frac{(^{46}Ti^{2+})_m+(^{47}Ti^{2+})_m}{(^{46}Ti^{2+})_s+(^{47}Ti^{2+})_s} \quad \text{Eq (S1)}$$

$$^{24}C_{corr}=(24Da)_{c\text{-}m}-(24Da)_m \quad \text{Eq (S2)}$$
